# Patient safety in ICUs: adverse events characterization and potential risk factors

**DOI:** 10.1186/cc9901

**Published:** 2011-03-11

**Authors:** L Zambon, R Daud-Gallotti, K Padilha, T Vasconcelos, N Inoue, F Rodrigues, L Tanigushi, I Velasco

**Affiliations:** 1 Clinics Hospital of University of São Paulo School of Medicine, São Paulo, Brazil; 2 University of São Paulo School of Nursing, São Paulo, Brazil; 3 University of São Paulo School of Medicine, São Paulo, Brazil

## Introduction

ICUs are complex settings, with critically ill patients submitted to invasive care, involving a multidisciplinary team, requiring urgent high-risk decision-making, taking place in an expensive structure with new technologies of increasing complexity. All these conditions facilitate the development of adverse events (AEs). We aimed to determine the occurrence of AEs in four tertiary academic ICUs in Brazil, disclosing their potential risk factors.

## Methods

This prospective cohort was conducted in four medical ICUs of a major academic, tertiary hospital in Brazil, enrolling all adult admissions during June to August 2009. AEs were identified by direct daily monitoring of medical and nursing rounds and chart review. Age, sex, APACHE II scores, length of stay (LOS), and the Nursing Activities Score (NAS) were also registered. The association with the occurrence of AEs was analyzed with logistic regression.

## Results

A total of 180 ICU admissions were included, regarding 176 patients (male/female: 86/90; age: 52.7 ± 1.8 years). The mean LOS, APACHE II scores and NAS were 10.0 ± 0.8 days, 15.7 ± 0.5 points and 69.0 ± 1.5%. Nearly 78% of the admissions (141 admissions) suffered 1,065 AEs. The most frequent AEs were: new dermatitis/pressure ulcers (195 events = 18.3% of events); hypoglycemic episodes not related to insulin use (HENI) (168 events = 15.8%); diagnostic/treatment failures (156 events = 14.6%); and drug AEs (195 events = 12.8%). Those four categories responded for 61.5% of all detected AEs. In the final logistic regression model, three independent variables remained as important risk factors for the occurrence of at least one AE: LOS >3 days, APACHE scores >13 points and NAS >70%, with adjusted OR estimates of 19.5, 3.4 and 3.3, respectively (*P *< 0.02).

## Conclusions

This prospective study was essential to identify the proportion of our ICU admissions affected by AEs, disclosing their nature. Our AE rates, affecting nearly 78% of admissions, were higher than those previously described. The direct observation of the ICUs contributed to those rates. Six out of 10 AEs corresponded to new cutaneous lesions, HENI, diagnostic/treatment failures and drug AEs. Length of stay, severity on admission and nursing workload were important risk factors for the occurrence of at least one AE.
